# Transcriptomic Analysis of Selenium-Induced Antioxidant Responses in Diploid, Triploid, and Tetraploid Pacific Oysters (*Crassostrea gigas*)

**DOI:** 10.3390/foods15122065

**Published:** 2026-06-07

**Authors:** Yousen Zhang, Cuiju Cui, Qihao Luo, Yuting Meng, Zan Li, Guohua Sun, Yanwei Feng, Xiaohui Xu, Jianmin Yang, Weijun Wang

**Affiliations:** 1 School of Fisheries, Ludong University, Yantai 264025, China; 19909024486@163.com (Y.Z.); cuicuiju@163.com (C.C.); lizanlxm@163.com (Z.L.); sgh_smile@163.com (G.S.); fywzxm1228@163.com (Y.F.); xxh83121@163.com (X.X.); ladderup@126.com (J.Y.); 2 Yantai KongTong Island Industrial Co., Ltd., Yantai 264000, China; 17854266371@163.com; 3 College of Fisheries and Life Science, Shanghai Ocean University, Shanghai 201306, China; mengyuting0611@163.com

**Keywords:** Pacific oyster, ploidy, selenium-enriched yeast, transcriptomic analysis, redox regulation, glutathione metabolism

## Abstract

Selenium-enriched yeast is an important organic selenium source for improving the nutritional value and physiological function of aquatic products. However, whether ploidy affects the molecular responses of Pacific oysters (*Crassostrea gigas*) under selenium supplementation conditions remains unclear. In this study, diploid, triploid, and tetraploid Pacific oysters were fed a selenium-enriched diet for 10 days. Hepatopancreas tissues were collected for antioxidant enzyme assays and transcriptomic analysis. The results showed that triploid oysters exhibited higher superoxide dismutase, glutathione peroxidase, and catalase activities, together with a relatively lower malondialdehyde level, suggesting stronger antioxidant defense under selenium supplementation conditions. Transcriptomic analysis revealed substantial numbers of differentially expressed genes across all groups compared, indicating pronounced ploidy-dependent transcriptional responses. Functional enrichment analysis showed that these genes were mainly associated with metabolic processes, oxidative stress responses, membrane components, endocrine-related pathways, lipid metabolism, and signal transduction. Several redox-related genes, including glutathione S-transferase family genes, CHAC1, superoxide dismutase family genes, and NRX1, showed ploidy-dependent expression patterns. In particular, triploid oysters appeared to rely more on enzymatic antioxidant defense and glutathione turnover, whereas tetraploid oysters showed a stronger tendency toward redox signal integration and homeostatic regulation. These findings provide new insights into ploidy-dependent selenium responses in Pacific oysters and offer a theoretical basis for the development and utilization of selenium-enriched oyster products.

## 1. Introduction

Selenium (Se) is an essential trace element for maintaining normal physiological functions in organisms. Its biological roles are primarily exerted through its incorporation into and participation in the functions of selenoproteins, such as glutathione peroxidases and thioredoxin reductases. Consequently, Se plays crucial roles in antioxidant defense, immune regulation, thyroid hormone metabolism, and the maintenance of cellular redox homeostasis [1,2,3]. Notably, the physiological effects of Se exhibit a distinct dose-dependent characteristic, and both deficiency and excessive intake can lead to metabolic disorders [4,5]. Therefore, screening safe, stable, and highly bioavailable natural Se sources constitutes a significant focus in the development and nutritional fortification of functional aquatic products.

In aquatic organisms, the efficiency of selenium utilization is closely associated with species-specific physiological and metabolic characteristics, which provides a biological basis for its accumulation and transformation in marine animals. As typical filter-feeding bivalves, oysters exhibit a strong capacity for accumulating and transforming mineral elements, making them an ideal candidate for developing functional foods of marine origin [6]. Studies have shown that Se in oysters predominantly exists in organic forms, such as selenomethionine (C_5_H_11_NO_2_Se), which generally possess high bioavailability. In Pacific oysters (*Crassostrea gigas*), selenomethionine can act as an efficient dietary Se source, promoting Se accumulation and retention in tissues, and contributing to improved antioxidant capacity and stress resistance through its involvement in selenium-containing protein synthesis [7]. This characteristic contributes to the high nutritional value of oysters and positions them as an important resource for developing Se-enriched aquatic products [8,9]. Therefore, from the perspectives of aquaculture and nutritional fortification, oysters possess considerable potential as carriers for Se-enriched products. A comprehensive understanding of the absorption, transformation, and utilization patterns of Se in oysters is of great importance for the targeted cultivation and quality enhancement of selenium-rich oysters. Moreover, elucidating the molecular mechanisms underlying selenium metabolism in oysters is essential for understanding how these physiological advantages are regulated at the transcriptional level.

In recent years, polyploid breeding technology has been widely applied in the genetic improvement and aquaculture production of oysters [10,11,12]. Among them, triploid oysters, whose reproductive capacity is inhibited, can allocate more resources to somatic growth and nutrient accumulation, resulting in faster growth rates and stable commercial traits. Consequently, they have become a primary focus in aquaculture production. Tetraploid oysters, serving as key parental material for producing triploid seedlings, play a crucial role in oyster breeding systems [13,14,15]. Although numerous studies have explored the differences in growth performance, reproductive characteristics, and environmental adaptability among oysters of different ploidies, research on nutrient metabolism, particularly the potential differences in Se absorption, transformation, utilization, and antioxidant regulatory mechanisms under Se supplementation conditions, remains limited [16,17].

From a molecular perspective, ploidy variation may influence gene dosage effects and transcriptional regulatory networks, thereby potentially altering selenium-associated metabolic and stress response pathways [18,19]. Previous research has demonstrated that Se intervention can significantly alter the expression of genes related to metabolism and antioxidant processes in oysters. Differentially expressed genes (DEGs) are primarily enriched in pathways associated with antioxidant defense, substance metabolism, and immune regulation, suggesting a broad impact of Se nutrition on the physiological status of oysters [20,21]. Meanwhile, transcriptomic studies on oysters of different ploidies indicate that ploidy changes can reshape multiple molecular pathways related to growth, metabolism, and stress response [14,20]. However, a systematic comparison is still lacking regarding whether oysters of different ploidies exhibit distinct transcriptional response patterns under uniform selenium nutritional intervention. In particular, it remains unclear whether selenium metabolism-related and antioxidant regulation-related pathways display ploidy-dependent regulatory characteristics. Transcriptome sequencing technology enables the comprehensive analysis of gene expression changes and functional pathway responses at the whole-genome level, providing a powerful tool for uncovering the Se response mechanisms in oysters of different ploidies [21,22].

Based on this, the present study employed diploid, triploid, and tetraploid Pacific oysters as the research materials. Under conditions of Se supplementation, we systematically compared the transcriptional responses among individuals of different ploidies by combining transcriptome sequencing. The analysis focused on identifying differentially expressed genes (DEGs) and functional pathways associated with Se metabolism, antioxidant defense, and related metabolic processes. The aim is to clarify the influence of ploidy differences on the selenium response strategies in oysters and their molecular regulatory basis. This study provides new insights into ploidy-dependent selenium responses and their underlying molecular mechanisms in Pacific oysters.

## 2. Materials and Methods

### 2.1. Experimental Materials and Design

Pacific oysters were used as the experimental material. Diploid, triploid, and tetraploid oysters were collected from the breeding base on Kongtong Island, Yantai, Shandong Province, China. Selected individuals had intact shells, high vitality, and were at the undeveloped gonadal stage. Prior to the experiment, oysters were acclimated for one week in a recirculating aquaculture system under controlled seawater conditions (temperature: 16 ± 1 °C; dissolved oxygen: 5.4 mg/L; pH: 8.1 ± 0.5; salinity: 30 ± 0.5 ppt). During acclimation, oysters were fed fresh *Nitzschia closterium f. minutissima* microalgae four times daily, and seawater was completely renewed once per day.

To evaluate ploidy-dependent physiological and transcriptomic responses under selenium supplementation conditions, three ploidy groups (diploid, triploid, and tetraploid) were established. Each ploidy group consisted of three independent culture tanks containing 20 oysters per tank, resulting in a total of 60 individuals per ploidy group. Selenium supplementation was conducted using selenium-enriched yeast (Angel Yeast Co., Ltd., Yichang, Hubei, China; selenium content: 2000 mg/kg), which was added to the daily diet at 1‰ of the total oyster body weight. All groups were maintained under the same selenium supplementation conditions throughout the experiment. The supplementation period lasted for 10 days, followed by 24 h of fasting prior to sampling.

After treatment, nine oysters were randomly selected from each ploidy group. Hepatopancreas tissues were dissected, immediately frozen in liquid nitrogen, and stored at −80 °C until further analysis. The samples were designated as Se_2N (diploid oysters under selenium supplementation), Se_3N (triploid oysters under selenium supplementation), and Se_4N (tetraploid oysters under selenium supplementation), respectively. These samples were subsequently used for antioxidant enzyme assays and transcriptome sequencing analysis.

### 2.2. Ploidy Verification

**Figure 1:**
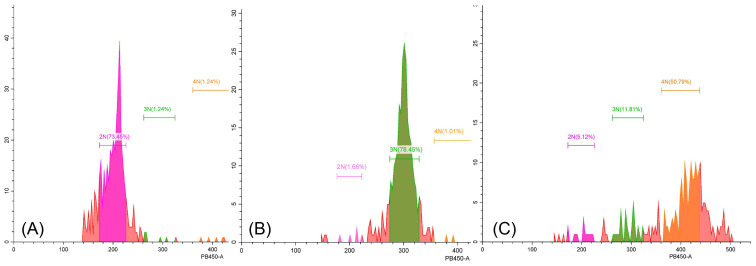
(**A**–**C**) Characteristic DNA content peaks representing diploid, triploid, and tetraploid oysters, respectively.

Ploidy verification for each oyster was performed by flow cytometry prior to tissue sampling following a previously described protocol in Pacific oysters [23]. Gill tissues were used to prepare single-cell suspensions in PBS, and nuclei were stained with DAPI for DNA content analysis. After incubation in the dark, samples were filtered and subsequently analyzed by flow cytometry to determine ploidy level based on fluorescence intensity (Figure 1).

### 2.3. Determination of Enzyme Activity Indicators

#### 2.3.1. Sample Preparation

Hepatopancreas tissue stored at −80 °C was weighed and homogenized in pre-cooled 0.9% saline solution at a 1:9 (*w*/*v*) ratio using a homogenizer on ice. The homogenate was centrifuged at 5000× *g* for 10 min at 4 °C, and the resulting supernatant was collected. This supernatant was used for measuring oxidative stress-related indicators, including catalase (CAT), malondialdehyde (MDA), superoxide dismutase (SOD), and glutathione peroxidase (GPx).

#### 2.3.2. Assay Procedures

Catalase (CAT) Activity: CAT activity was determined using a commercial assay kit (Nanjing Jiancheng Bioengineering Institute, Nanjing, China) following the manufacturer’s instructions. The assay is based on the decomposition rate of H_2_O_2_, which was monitored by measuring the decrease in absorbance at 240 nm using a microplate spectrophotometer. Briefly, tissue homogenate supernatant was added to the reaction mixture containing hydrogen peroxide substrate, and the absorbance change per minute was recorded. The assays were conducted in 96-well microplates with three technical replicates for each sample. CAT activity was expressed as units per milligram of protein (U/mg prot).

Malondialdehyde (MDA) Content: MDA content was measured using a commercial thiobarbituric acid (TBA) assay kit (Nanjing Jiancheng Bioengineering Institute, Nanjing, China) according to the manufacturer’s protocol. Briefly, the sample supernatant was mixed with TBA working reagent and incubated in a 95 °C water bath for 15 min to form the chromogenic product. After cooling to room temperature, the absorbance was measured at 532 nm using a microplate spectrophotometer. All assays were performed in 96-well plates with three technical replicates. The MDA concentration was expressed as nanomoles per milligram of protein (nmol/mg prot).

Superoxide Dismutase (SOD) Activity: SOD activity was determined using a commercial assay kit (Nanjing Jiancheng Bioengineering Institute, Nanjing, China) based on the inhibition of nitroblue tetrazolium (NBT) reduction by superoxide anions generated in the reaction system. Sample supernatant was added to the reaction mixture according to the manufacturer’s instructions, and absorbance was measured at 550 nm using a microplate spectrophotometer. One unit of SOD activity was defined as the amount of enzyme causing 50% inhibition of the reaction rate. All measurements were conducted in triplicate in 96-well microplates. Results were expressed as units per milligram of protein (U/mg prot).

Glutathione Peroxidase (GPx) Activity: GPx activity was assayed using a commercial kit (Nanjing Jiancheng Bioengineering Institute, Nanjing, China) according to the manufacturer’s protocol. The assay measures the oxidation rate of reduced glutathione catalyzed by GPx during the reduction in H_2_O_2_. The absorbance change was monitored spectrophotometrically using a microplate reader. All assays were conducted in 96-well plates with three technical replicates per sample. GPx activity was expressed as units per milligram of protein (U/mg prot).

#### 2.3.3. Data Processing

All experiments were conducted using three independent biological replicates, and each biological replicate was measured in triplicate as technical replicates. Statistical analysis was performed using SPSS Statistics 22 software (IBM Corp., Armonk, NY, USA). Differences among treatment groups were analyzed using one-way analysis of variance (ANOVA), and statistical significance was defined as *p* < 0.05.

### 2.4. Total RNA Extraction, cDNA Library Construction, and Sequencing

Total RNA was extracted from oyster hepatopancreas tissue using TRIzol reagent (Invitrogen, Carlsbad, CA, USA) according to the manufacturer’s instructions, which is a widely used acid guanidinium thiocyanate–phenol–chloroform extraction method [24]. Briefly, approximately 50–100 mg of tissue was homogenized in TRIzol reagent, followed by phase separation with chloroform, RNA precipitation with isopropanol, and washing with 75% ethanol. RNA integrity and purity were rigorously assessed using an Agilent 2100 Bioanalyzer (Santa Clara, CA, USA).

The library construction method followed a referenced protocol. Briefly, for each experimental group, RNA from three biological replicates was pooled in equal amounts, and this pooling process was repeated to obtain three independent pooled RNA samples per ploidy group. Therefore, three independent pooled RNA libraries were constructed for each ploidy group, facilitating subsequent differential expression analysis. After the library quality was verified, high-throughput sequencing was performed on the Illumina NovaSeq 6000 platform.

### 2.5. Transcriptome Sequencing Data Processing and Analysis

Raw sequencing reads were processed to remove adapter sequences, reads containing poly-N sequences, and low-quality reads, yielding high-quality clean data. The Q20, Q30 scores, and GC content of the clean data were calculated. All subsequent analyses were based on the clean data. The reference genome index was built using HISAT2 v2.0.5, and the paired-end clean reads were aligned to the reference genome.

Raw sequencing reads were processed to remove adapter sequences, poly-N reads, and low-quality reads, yielding high-quality clean data. Quality metrics, including Q20, Q30 scores, and GC content, were calculated to evaluate sequencing quality. All downstream analyses were performed based on clean reads. This quality control strategy follows standard RNA-seq preprocessing workflows widely adopted in transcriptomic studies [25].

The reference genome index was built using HISAT2 v2.0.5, and paired-end clean reads were aligned to the Pacific Oyster reference genome. HISAT2 is a fast and sensitive spliced aligner that is widely applied for RNA-seq read mapping in eukaryotic transcriptome studies due to its high alignment efficiency and accuracy [26].

### 2.6. Differential Expression Gene Analysis

Based on the alignment results, differential expression analysis between sample groups was performed using the DESeq2 software. Three comparisons were conducted: Se_3N vs. Se_2N, Se_4N vs. Se_2N, and Se_4N vs. Se_3N, to assess the transcriptional response differences among oysters of different ploidy under selenium treatment. Differentially expressed genes (DEGs) were identified using the criteria of adjusted *p*-value ≤ 0.05 and |log_2_(fold change)| ≥ 1. Subsequently, the identified DEGs were mapped to Gene Ontology (GO) functional categories and the Kyoto Encyclopedia of Genes and Genomes (KEGG) pathway database for functional enrichment analysis. Clustering analysis was also performed on DEGs with significant expression patterns to reveal the potential molecular regulatory mechanisms underlying the response to selenium in oysters of different ploidy.

### 2.7. Data Availability

The RNA-seq data generated in this study have been deposited in the NCBI Sequence Read Archive (SRA) under BioProject accession number PRJNA1470089. The data will be publicly available after the embargo period and can be accessed at https://www.ncbi.nlm.nih.gov/sra/PRJNA1470089 (accessed on 6 January 2026).

## 3. Results

### 3.1. Effects of Selenium Yeast on Enzyme Activity in Pacific Oysters

**Figure 2:**
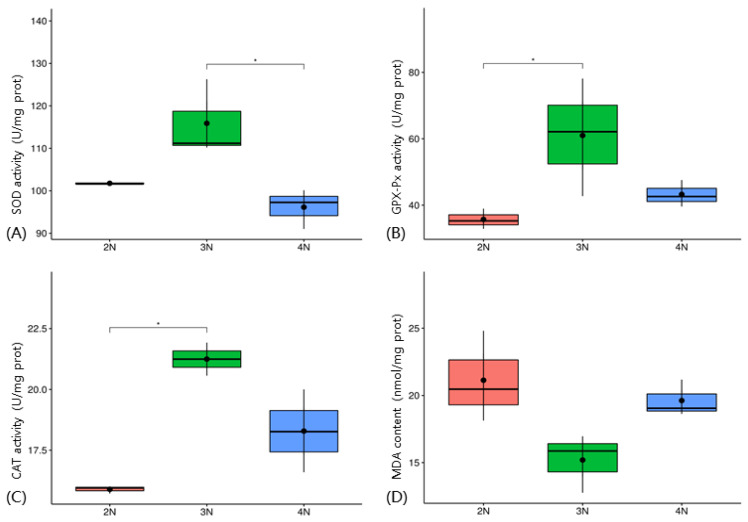
Comparison of antioxidant enzyme activities and MDA content in oysters of different ploidy. (**A**) Superoxide dismutase (SOD) activity; (**B**) Glutathione peroxidase (GPx-Px) activity; (**C**) Catalase (CAT) activity; (**D**) Malondialdehyde (MDA) content. * *p* < 0.05 indicates a significant difference between groups.

As shown in Figure 2, Pacific oysters of different ploidy exhibited distinct variations in antioxidant enzyme activities and lipid peroxidation levels. The activity of superoxide dismutase (SOD, Figure 2A) was highest in triploid oysters, followed by diploids, and lowest in tetraploids, with the activity in 3N being significantly higher than that in 4N (*p* < 0.05). The activity of glutathione peroxidase (GPx, Figure 2B) showed a similar trend among ploidies, with 3N being significantly higher than 2N (*p* < 0.05), while 4N showed intermediate levels without significant differences. Catalase (CAT, Figure 2C) activity was also highest in 3N, significantly exceeding that in 2N (*p* < 0.05), whereas 4N again showed intermediate levels without significant differences. In contrast, malondialdehyde (MDA, Figure 2D), a product of lipid peroxidation, showed an opposite trend to antioxidant enzyme activities. MDA content was highest in the 2N group, lowest in the 3N group, and intermediate in the 4N group; however, no statistically significant differences were observed among groups.

### 3.2. Assembly of Sequencing Data

The quality of transcriptome sequencing data from the samples was generally high (Table 1). The numbers of clean reads for diploid, triploid, and tetraploid samples were 43,182,512, 44,689,791, and 44,264,606, respectively, while the raw reads were 44,736,770, 45,901,000, and 45,497,693, respectively. The mapping rates of reads to the reference genome for each sample were 64.18%, 64.66%, and 63.81%, respectively, and the uniquely mapped rates were 60.20%, 60.65%, and 59.72%, respectively. Furthermore, the GC contents for diploid, triploid, and tetraploid samples were 43.45%, 43.58%, and 43.40%, respectively. The Q20 and Q30 scores for all samples exceeded 97.52% and 92.98%, respectively, indicating that the sequencing data were of good quality and suitable for subsequent analysis.

### 3.3. Analysis of Differentially Expressed Genes

**Figure 3:**
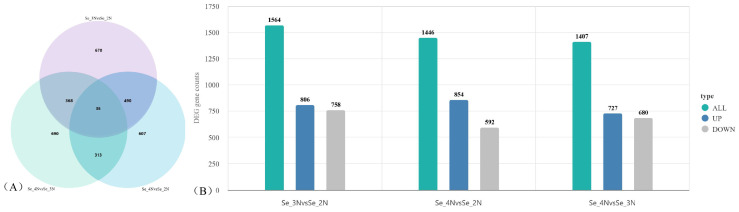
(**A**) Venn diagram of the number of DEGs. (**B**) Number of up- or down-regulated DEGs between different comparison groups.

Based on the transcriptome sequencing results, oysters of different ploidy exhibited notable differences in gene expression under the same selenium yeast intervention. As shown in Figure 3A, the three comparison groups shared 36 core DEGs. In addition, 490 DEGs were shared between Se_3N vs. Se_2N and Se_4N vs. Se_2N, 369 between Se_4N vs. Se_2N and Se_4N vs. Se_3N, and 313 between Se_3N vs. Se_2N and Se_4N vs. Se_3N. The numbers of unique DEGs were 670, 607, and 690 in Se_3N vs. Se_2N, Se_4N vs. Se_2N, and Se_4N vs. Se_3N, respectively.

Further analysis (Figure 3B) showed that 1564, 1446, and 1407 DEGs were identified in Se_3N vs. Se_2N, Se_4N vs. Se_2N, and Se_4N vs. Se_3N, respectively. Among them, 806, 854, and 727 were upregulated, while 758, 592, and 680 were downregulated, respectively.

### 3.4. Functional Annotation and Enrichment Analysis of DEGs

#### 3.4.1. GO Enrichment Analysis

**Figure 4:**
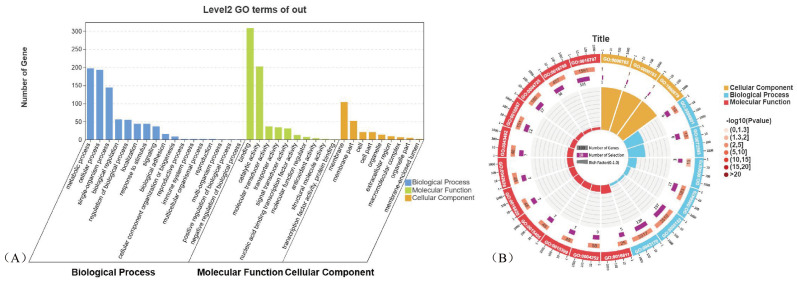
(**A**) Bar chart of GO enrichment secondary classification. (**B**) Bubble chart of GO enrichment. (**B**) GO:0006801: superoxide metabolic process; GO:0072593: reactive oxygen species metabolic process; GO:0008152: metabolic process; GO:0016787: hydrolase activity; GO:0004252: serine-type endopeptidase activity; GO:0016887: ATPase activity; GO:0004725: protein tyrosine phosphatase activity; GO:0016788: hydrolase activity, acting on ester bonds; GO:0016811: hydrolase activity, acting on carbon-nitrogen (but not peptide) bonds; GO:0006470: protein dephosphorylation; GO:0043170: macromolecule metabolic process; GO:0015399: primary active transmembrane transporter activity; GO:0015405: P-P-bond-hydrolysis-driven transmembrane transporter activity; GO:0016820: hydrolase activity, acting on acid anhydrides; GO:0042626: ATPase-coupled activity; GO:0043492: ATPase-coupled proton transport; GO:0000270: peptidoglycan metabolic process; GO:0000782: nucleosome core particle; GO:0000783: nuclear nucleosome DNA binding; GO:1990879: CENP-A containing nucleosome.

Gene Ontology (GO) enrichment analysis of the differentially expressed genes revealed that their functions could be categorized into three main classes: Biological Process (BP), Cellular Component (CC), and Molecular Function (MF) (Figure 4). Overall, the DEGs were primarily enriched in categories related to fundamental biological activities such as metabolic processes, binding functions, and membrane structures. In terms of Biological Process, in addition to broad involvement in metabolic and cellular processes, the DEGs were significantly associated with biological regulation, response to stimulus, and signal transduction. Regarding Molecular Function, besides binding and catalytic activities, functions related to signal transduction and transcriptional regulation were also prominent. For Cellular Component, DEGs were mainly concentrated in structures such as membranes and membrane parts, organelles, and the extracellular region. Collectively, these results indicate that the DEGs are primarily involved in functional categories such as metabolic processes, membrane structure composition, response to stimuli, and signal transduction, suggesting widespread functional response differences among oysters of different ploidy under selenium yeast nutritional intervention.

#### 3.4.2. KEGG Pathway Enrichment Analysis

**Figure 5:**
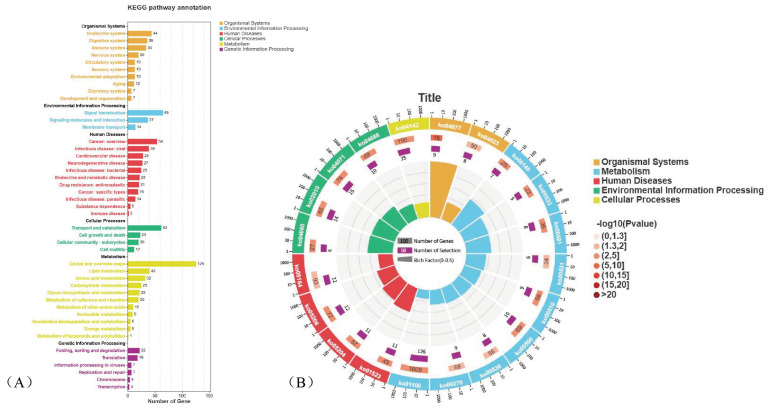
(**A**) Statistics of KEGG enrichment entries. (**B**) Bubble chart of KEGG enrichment. (**B**) KO:04977: Vitamin digestion and absorption; KO:00140: Steroid hormone biosynthesis; KO:00533: Glycosaminoglycan biosynthesis—keratan sulfate; KO:01523: Antifolate resistance; KO:00591: Linoleic acid metabolism; KO:04060: Cytokine-cytokine receptor interaction; KO:02010: ABC transporters; KO:04071: Sphingolipid signaling pathway; KO:05204: Chemical carcinogenesis—DNA adducts; KO:00531: Glycosaminoglycan degradation; KO:05206: MicroRNAs in cancer; KO:00510: N-Glycan biosynthesis; KO:04623: Cytosolic DNA-sensing pathway; KO:00590: Arachidonic acid metabolism; KO:04668: TNF signaling pathway; KO:00830: Retinol metabolism; KO:00270: Cysteine and methionine metabolism; KO:04142: Lysosome; KO:05164: Influenza A; KO:01100: Metabolic pathways.

Kyoto Encyclopedia of Genes and Genomes (KEGG) pathway enrichment analysis revealed that the DEGs were mainly enriched in six major functional categories (Figure 5A). Among these, pathways related to metabolism contained the highest number of enriched genes, followed by categories such as genetic information processing and membrane transport. Further analysis of enrichment significance (Figure 5B) showed that pathways related to the endocrine system (e.g., Insulin signaling pathway), lipid metabolism (e.g., Fatty acid degradation), and signal transduction (e.g., MAPK signaling pathway) were highly enriched (−log_10_(*p*-value) > 5). These results indicate that the significantly enriched pathways are primarily concentrated in processes related to the endocrine system, lipid metabolism, and signal transduction, suggesting their potential involvement in the differential transcriptional responses of oysters with different ploidy to selenium yeast nutritional intervention.

## 4. Discussion

Under the nutritional intervention of selenium yeast, Pacific oysters of different ploidy exhibited distinct antioxidant physiological responses and transcriptional expression profiles. Triploid oysters showed higher activities of SOD, GPx, and CAT, along with relatively lower MDA levels, suggesting a potentially enhanced antioxidant defense capacity under this treatment. Combined with transcriptome analysis, the observed differences in expression patterns of genes related to selenium metabolism, redox homeostasis, and antioxidant defense among ploidy groups indicate that ploidy variation may influence the molecular response of oysters to selenium intervention. Based on this, the redox regulation characteristics of oysters with different ploidy under selenium yeast treatment are discussed below, integrating key differentially expressed genes and associated pathways.

### 4.1. Ploidy-Dependent Regulatory Mechanisms of Genes Related to Glutathione Metabolism

#### 4.1.1. Isoform Differences in the GST Gene Family and Their Ploidy Response Characteristics

In this study, genes of the Glutathione S-transferase (GST) family, such as GSTO1, GST-8, GSTT1, and GSTN, were significantly downregulated in comparisons between different ploidies. This suggests that ploidy differences may affect the isoform-specific functional division within glutathione metabolism-related pathways, rather than simply inducing a globally enhanced antioxidant response. Previous studies have shown that GSTs, especially Omega class members, play important roles in redox regulation and participate in the adaptive response of organisms to oxidative stress and peroxide stimulation [27,28]. Therefore, changes in the expression of GST family genes are generally considered important molecular markers of cellular antioxidant regulation.

Notably, GSTO1 was downregulated in both the Se_3N vs. Se_2N and Se_4N vs. Se_2N comparisons. This result suggests that, under selenium nutritional intervention, oysters with higher ploidy may not primarily rely on the classic antioxidant pathway mediated by GSTO1. Instead, they may maintain cellular homeostasis through the coordinated regulation of other GST isoforms, SOD family members, and related redox regulatory molecules, which is considered an important antioxidant adaptation strategy in aquatic organisms under environmental or nutritional stress [29,30]. Accordingly, this expression pattern is more likely to reflect a functional restructuring and compensatory regulation of antioxidant pathways rather than a simple reduction in antioxidant capacity, as adaptive coordination among antioxidant systems has also been observed in marine bivalves under stress conditions [31,32]. In this context, the downregulation of certain GST isoforms may help reduce excessive glutathione consumption, thereby preserving intracellular glutathione for other critical redox regulatory processes.

This speculation is consistent with previous conclusions that the GST gene family exhibits isoform-specific expression differences across different species, tissues, and stress conditions [33]. Therefore, the downregulation of GSTO1 and related GST genes in this study is more likely to suggest that oysters of different ploidy employ differentiated glutathione utilization strategies under selenium treatment. This involves optimizing the functional division of GST isoforms to improve the efficiency of antioxidant resource allocation, thereby adapting to redox regulation demands under different ploidy backgrounds.

#### 4.1.2. Regulatory Characteristics of the CHAC1 Gene in Ploidy-Dependent Selenium Response

CHAC1 showed a distinct differential expression pattern among oysters of different ploidy: upregulated in Se_3N vs. Se_2N, but downregulated in Se_4N vs. Se_3N. This indicates that the expression of CHAC1 is not only associated with exogenous selenium treatment but may also be significantly influenced by ploidy differences. CHAC1 encodes a member of the γ-glutamylcyclotransferase family, which can specifically degrade glutathione (GSH) and plays a crucial role in regulating cellular redox homeostasis. Previous studies have shown that upregulation of CHAC1 is typically associated with GSH depletion, increased oxidative pressure, and activation of related stress pathways [34,35].

The upregulation of CHAC1 in Se_3N vs. Se_2N may reflect a higher GSH turnover demand in triploid oysters under selenium nutritional intervention. Combined with the higher activities of SOD, GPx, and CAT observed in triploids in this study, it can be inferred that triploids may more effectively buffer oxidative pressure by accelerating GSH renewal in synergy with antioxidant enzymes, thereby maintaining a stronger antioxidant defense state. In other words, CHAC1 upregulation in triploids may not necessarily signify enhanced oxidative damage alone; it is more likely to represent a dynamic regulatory process that matches high antioxidant metabolic activity. Previous research has indicated that CHAC1 upregulation under exogenous oxidative stress participates in the oxidative stress response by promoting GSH degradation [36].

In contrast, the downregulation of CHAC1 in Se_4N vs. Se_3N suggests that tetraploid oysters may be more inclined to inhibit excessive GSH breakdown to maintain a stable intracellular reducing environment. Previous studies have shown that CHAC1 downregulation helps mitigate oxidative damage and buffer stress-related cellular harm [37,38]. Furthermore, other studies have shown that CHAC1 functions as a glutathione-degrading enzyme involved in cellular redox regulation, while maintenance of glutathione levels is closely associated with enhanced antioxidant capacity and oxidative stress alleviation in aquatic animals [34,39]. Therefore, the ploidy-dependent expression pattern of CHAC1 observed in this study may reflect differences in glutathione regulation among oysters of different ploidy under selenium treatment. Triploid oysters may tend to promote GSH turnover to meet antioxidant demands, whereas tetraploid oysters may be more inclined to preserve GSH reserves to support a relatively stable redox state.

### 4.2. Ploidy-Dependent Expression Characteristics of the SOD Gene Family in the Antioxidant Enzyme System

Superoxide dismutase (SOD) is the first line of defense against superoxide anion radicals and plays a key role in maintaining cellular redox homeostasis. The SOD family in oysters exhibits high genetic diversity, including intracellular Cu/Zn-SOD, extracellular Cu/Zn-SOD, and Mn-SOD isoforms [40]. Different isoforms often display specific expression patterns in response to environmental stress. Therefore, expression differences in SOD family genes can serve as important molecular clues for deciphering the antioxidant regulation strategies of oysters with different ploidy.

This study showed that multiple genes related to cytosolic Cu/Zn-SOD, such as SODC, SOD1, and SOD3, exhibited an overall upregulated trend in triploids and tetraploids compared to diploids. This result is consistent with the higher SOD enzyme activity in triploids, indicating that higher-ploidy individuals may possess a stronger ability to scavenge superoxide anions under selenium treatment, thereby enhancing their basal antioxidant defense level. Previous studies have also noted that in oysters, SOD family genes can be significantly induced under temperature, salinity, or other oxidative stress conditions, participating in ROS scavenging and stress adaptation processes [41]. Therefore, the upregulation of SOD-related genes observed here may reflect the adaptive regulation of higher-ploidy oysters to oxidative pressure in the context of selenium treatment.

Furthermore, the upregulation of the extracellular Cu/Zn-SOD member SOD3 in the Se_4N vs. Se_2N comparison is particularly noteworthy. Extracellular SOD plays an important role in maintaining the homeostasis of the extracellular oxidative environment. Its enhanced expression suggests that the antioxidant regulation in tetraploid oysters may not be limited to intracellular ROS scavenging but could extend to the extracellular microenvironment level. While extracellular SOD-related homologs may not always possess typical enzymatic activity, they are still considered to participate in oxidative stress defense and immune regulation [40,42]. Based on the results of this study, it can be speculated that tetraploid oysters may possess a broader scope of antioxidant regulation under selenium nutritional intervention, not solely relying on classic intracellular enzymatic scavenging pathways.

Concurrently, previous studies have indicated that oysters of different ploidy exhibit differences in antioxidant enzyme activities and oxidative damage levels under environmental stress, with triploids generally showing stronger antioxidant potential [43]. The results obtained in this study under selenium treatment are largely consistent with this trend, further supporting the notion that ploidy differences may influence the overall oxidative stress response capacity of oysters by modulating the expression patterns of the SOD family.

### 4.3. Expression Changes in Thioredoxin-Related Genes Reveal Regulatory Hierarchy Differences

Beyond glutathione metabolism and the classic antioxidant enzyme system, thioredoxin-related pathways are also important components in maintaining cellular redox homeostasis. In this study, the significant upregulation of NRX1(nucleoredoxin 1) in Se_4N vs. Se_3N suggests that tetraploid oysters may rely more on thioredoxin-related regulatory mechanisms under selenium nutritional intervention. NRX1 belongs to the non-classical thioredoxin family. In addition to participating in redox reactions, it can regulate various redox-sensitive signaling pathways, such as Wnt/β-catenin and MAPK pathways, thereby playing an upstream regulatory role in maintaining cellular homeostasis and environmental adaptation [44,45].

The upregulation of NRX1 in tetraploids suggests that their antioxidant regulation pattern may not primarily depend on enhanced direct ROS scavenging. Compared with triploids, which exhibited relatively higher antioxidant enzyme activities, tetraploids showed a more pronounced upregulation of NRX1, implying a potential involvement of redox signaling and cellular homeostasis regulation in their response to selenium supplementation. As NRX1 is known to participate in cellular redox regulation, its elevated expression may reflect differences in redox regulatory responses among ploidy groups. This pattern, together with the expression profiles of GST family members and SOD-related pathways, suggests that oysters of different ploidy may exhibit distinct antioxidant response characteristics under the same selenium conditions.

From a ploidy physiology perspective, tetraploid individuals may possess more complex gene dosage effects and regulatory networks. Therefore, their response to external stimuli may depend more on high-level signal coordination mechanisms. Based on the findings of this study, it can be inferred that the upregulation of NRX1helps tetraploids maintain antioxidant capacity while avoiding the over-enhancement of ROS scavenging processes and excessive GSH consumption, thereby achieving a more stable and sustained redox balance. Overall, the differential expression of NRX1 further suggests that as ploidy increases, the redox regulation mode of oysters under selenium treatment may shift from a “metabolic antioxidant” pattern towards a “signaling-based homeostasis regulation” pattern.

### 4.4. Integrated Model of Redox Regulation in Pacific Oysters of Different Ploidy Under Selenium Yeast Supplementation

**Figure 6:**
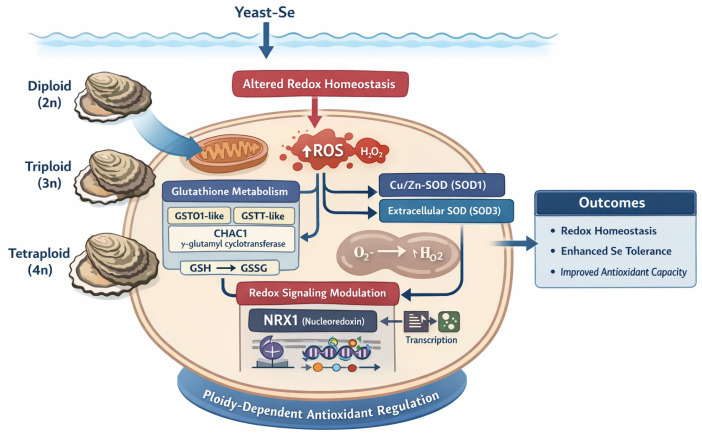
An integrated model of redox regulation in Pacific oysters of different ploidy under selenium yeast supplementation. Se represents selenium-enriched yeast. ROS, reactive oxygen species; GSH, glutathione; GSSG, oxidized glutathione; GST, glutathione S-transferase; GSTO1-like, glutathione S-transferase omega 1-like; GSTT-like, glutathione S-transferase theta-like; CHAC1, ChaC glutathione-specific γ-glutamylcyclotransferase 1; SOD1, Cu/Zn-superoxide dismutase; SOD3, extracellular superoxide dismutase; NRX1, nucleoredoxin 1; O_2_^−^, superoxide anion; H_2_O_2_, hydrogen peroxide.

Integrating the changes in antioxidant enzyme activities, differentially expressed gene analysis, and functional enrichment results, this study proposes an integrated model of redox regulation in Pacific oysters of different ploidy under selenium yeast supplementation (Figure 6). As illustrated in Figure 6, exogenous selenium yeast treatment may influence the balance between reactive oxygen species (ROS) generation and scavenging, thereby triggering coordinated responses involving the glutathione metabolism pathway, SOD enzyme system, and thioredoxin-related regulatory network. In this model, key differentially expressed genes, including GST family members (GSTO1-like, GSTT-like), CHAC1, SOD1/SOD3, and NRX1, are explicitly mapped to their corresponding functional modules, highlighting their potential roles in regulating redox homeostasis under different ploidy backgrounds.

Combining physiological indicators with transcriptomic evidence, triploid oysters, characterized by higher SOD, GPx, and CAT activities together with lower MDA levels, may possess a relatively stronger capacity to alleviate oxidative stress through enhanced enzymatic antioxidant activity and glutathione turnover. In contrast, the more pronounced upregulation of NRX1 in tetraploid oysters suggests a greater reliance on redox signaling and homeostatic regulation to maintain cellular balance. These results, as summarized in Figure 6, suggest that under selenium yeast supplementation, Pacific oysters of different ploidy exhibit ploidy-dependent response patterns, with triploids tending toward more direct enzymatic antioxidant defense, whereas tetraploids appear to rely more on redox regulatory coordination and homeostasis maintenance.

## 5. Conclusions

Under identical selenium yeast nutritional intervention, Pacific oysters of different ploidy exhibited distinct antioxidant physiological responses and transcriptional expression profiles. Triploid oysters showed higher activities of SOD, GPx, and CAT, together with lower MDA levels, indicating a stronger enzymatic antioxidant capacity under selenium supplementation. Transcriptomic analysis revealed that differentially expressed genes were mainly enriched in pathways related to antioxidant defense, substance metabolism, immune regulation, and signal transduction.

Overall, the results suggest that oysters of different ploidy adopt distinct redox regulation strategies in response to selenium yeast. Triploids tend to rely more on direct enzymatic antioxidant defense, whereas tetraploids may emphasize redox signaling coordination and cellular homeostasis maintenance. These findings highlight ploidy-dependent differences in selenium-responsive antioxidant regulation and provide new insights into the functional adaptation of polyploid Pacific oysters.

This study is limited to transcriptomic-level analysis and physiological indicators, without direct functional validation of key genes or proteins. Future studies should incorporate proteomic and functional experiments to further verify the roles of candidate genes (e.g., GST family members, CHAC1, and NRX1) and to clarify the underlying regulatory mechanisms in greater detail.

## Figures and Tables

**Table 1:** Summary statistics of transcriptome sequencing and average reads mapped to the diploid, triploid, and tetraploid oyster genomes.

Sample	2N	3N	4N
Clean reads	43,182,512	44,689,791	44,264,606
Raw reads	44,736,770	45,901,000	45,497,693
Mapped reads	27,731,707	28,886,310	28,252,637
Mapping rate (%)	64.18	64.66	63.81
Uniquely mapped reads	26,013,031	27,096,093	26,436,269
Uniquely mapped rate (%)	60.20	60.65	59.72
G/C content (%)	43.45	43.58	43.40
% ≥ Q20	97.52	97.63	97.62
% ≥ Q30	92.98	93.30	93.23

## Data Availability

The original contributions of this study are presented in this article. Further inquiries can be directed to the corresponding author.

## Abbreviations

The following abbreviations are used in this manuscript:

Catalase	CAT
Malondialdehyde	MDA
Superoxide Dismutase	SOD
Glutathione Peroxidase	GPx
